# Physical Activity Experiences of South Asian Migrant Women in Western Australia: Implications for Intervention Development

**DOI:** 10.3390/ijerph19063585

**Published:** 2022-03-17

**Authors:** Alexis Pullia, Zakia Jeemi, Miguel Reina Ortiz, Jaya A. R. Dantas

**Affiliations:** 1 Curtin School of Population Health, Curtin University, Perth 6102, Australia; alexispullia123@gmail.com (A.P.); zakia.jeemi@curtin.edu.au (Z.J.); 2 College of Public Health, University of South Florida, Tampa, FL 33612, USA; miguelreina@usf.edu

**Keywords:** South Asian, migrants, women, physical activity

## Abstract

The benefits of physical activity are widely recognised; however, physical activity uptake remains low in South Asian populations. South Asian migrant women face health risks as they adapt to new cultures, and these risks are often intensified through their limited participation in physical activity as one of the behaviours that promote positive health outcomes. Three focus group discussions with sixteen South Asian migrant women aged between 33 and 64 years, with a median age of 48 years and who live in Western Australia, were conducted. Thematic analysis of the transcribed qualitative data was completed to explore and uncover South Asian women’s experiences with physical activity, as well as their motivation, beliefs, attitudes, and knowledge about physical activity. Five major themes emerged after coding and analysing the data. The themes included the women’s knowledge of physical activity, their general attitudes and beliefs surrounding physical activity, the advantages and disadvantages of participation in physical activity, their experiences with physical activity, and the barriers, challenges, and facilitators surrounding physical activity. Recommendations are proposed to increase physical activity among this group to improve overall health and wellbeing and implications for intervention development are discussed.

## 1. Introduction

The number of foreign-born, permanent residents in Australia has continued to increase, as historically, more people migrate to then emigrate from the country [1]. In 2020, there were more than 7.6 million migrants living in Australia [1]. The rise in migration to Australia has only increased Australia’s diverse population, both culturally and linguistically [2]. Migration from South Asia, especially India to Australia, has increased in the last decade when compared to other dominant migrating populations, such as those from the United Kingdom and Europe [3]. In fact, the five countries with the highest immigration growth rates between 2006 and 2016 are Nepal (27.8%), Pakistan (13.2%), Brazil (12.1%), India (10.7%), and Bangladesh (8.9%). That is, 80% of these countries (four of these five countries) are in South Asia [3]. Furthermore, as of June 2020, India and Sri Lanka rank second and tenth, respectively, in the ranking of the Australian population by the top ten countries of birth [1]. It has been projected that by 2031, Asian populations will make up seven to nine percent of Australia’s population [4].

### 1.1. Gender and Migration

Historically, it has been recognised that women and men have different social and cultural roles [5,6]. During migration, varying social and cultural roles of men and women negatively influence migrating women. Gender acts as a socio-cultural influence upon the resettlement of migrants globally [6]. Typically, males are regarded as highly skilled professionals that add value to society, while women are expected to reconstruct their family’s domestic life [6]. This negatively impacts women’s abilities to cope with community structures in their host country [6] and exacerbates the marginalisation of migrant women in society when compared to their male counterparts [7]. This process, in turn, impedes the ability of the newly migrated women to reinvent their gender identities [5,6]. Women generally experience more inequalities than men in relation to integration outcomes [8]. Jarallah and Baxter [9], explored the association between gender and psychological distress among an Australian population of humanitarian entrants and found that women reported significantly higher distress than men.

Overall, it has been shown that gender-specific measures, interventions, and programs are pertinent to female migrants’ arrivals [5,6,8]. Cheung and Phillimore [8], argue that by creating gender-specific measures, it can be ensured that migrant women and children are embedded in society. With Australia being an immigrant nation [5], it is the country’s duty to call upon public policymakers to assist migrant women with finding their voice through proper integration in their country of resettlement. Previous research has identified that explicit gender-sensitive changes in national policy and availability of support groups are some ways that women can become more active in society during resettlement [6,8].

### 1.2. Improving Health through Physical Activity

A global epidemiological transition is affecting both developed and developing countries, leading to an increase in the burden of disease attributable to non-communicable diseases [10]. Individuals are living increasingly sedentary lifestyles, which have significant impacts on the health and wellbeing of individuals and populations [11]. When the increase of chronic diseases and sedentary lifestyles are combined, there is an accumulated risk for negative health outcomes [12,13]. Thus, it is reasonable to say that there is a substantial public health concern for the negative health outcomes associated with both low levels of physical activity and a sedentary life [11,12,14].

The impacts of both health-related changes are depicted globally in the leading causes of death worldwide, specifically cardiovascular disease, cancer, respiratory disease, diabetes, and dementia [15]. Notably, in Australia, coronary heart disease (CHD), dementia, cerebrovascular disease, lung cancer, and chronic obstructive pulmonary dis-ease are the top five leading causes of death [16]. Risk factors that contribute to the leading causes of death globally and in Australia include unhealthy diet, physical inactivity, and tobacco use [12].

Addressing physical inactivity has been found to be the most economical and effective manner of preventing the leading causes of death, especially cardiovascular disease [11,12,17,18]. Low levels of exercise and insufficient moderate and vigorous levels of physical activity [10,11] are modifiable through behaviour change that begins with minimal financial investment and time [11]. For decades, on a global level, the benefits of sport and physical activity have been observed and used for the empowerment of women and children, social justice, and to achieve gender equality [19]. Today, in developed countries, physical activity is present in government policies due to its significance related to health care systems and the economy [20]. More specifically, Bailey et al. [17], discusses the human capital model that combines the benefits of physical activity in public policy to support the fact that physical activity has the capacity to deliver valuable returns in the form of physical, emotional, individual, social, intellectual, and financial capital.

The Australian Government Department of Health provides national guidelines and recommendations to address sedentary behaviour and to encourage physical activity [21]. Despite these recommendations, Australians are not fulfilling their weekly or daily physical activity requirements. According to the Australian Institute of Health and Welfare, data from the 2017–18 National Health Survey (2017–2018) show 55% of adults did not participate in sufficient physical activity with women more frequently found to be insufficiently active (59%) when compared to men (50%) [22]. This poses a public health concern because research has demonstrated that risk factors related to obesity change very little from childhood to adulthood [17].

### 1.3. Physical Activity Interventions for South Asian Women and Immigrants

Previous research has identified that there are various barriers that South Asian women face when participating in physical activity [23,24,25]. Babakus and Thompson [23] reported that these difficulties include both cultural and structural barriers. Namely, South Asian women justify that partaking in physical activity takes time away from their families and communities and is described as a selfish act. This is further compounded by gender roles within South Asian culture which often state that a woman’s focus should be on family and to perform domestic duties [23,25]. Thus, women do not receive the necessary support needed to be physically active [25]. Cultural and structural barriers, inappropriate interventions, and facilities have acted as barriers to the participation of this population in physical activity [23]. Thus, using physical activity as a protective measure against non-communicable chronic diseases has been extremely low in this population [23,24,25]. It has also been noted that a lack of education and understanding surrounding physical activity, the recommended levels, and its benefits inhibit one’s choice and ability to successfully engage in physical activity [23].

South Asians, including women, are significantly burdened by chronic diseases [26,27,28]. When compared to their Caucasian counterparts, South Asians face CHD, diabetes, abdominal adiposity, and corresponding complications at younger ages [24,29]. A study of 16,287 urban South Asian adults reported on the prevalence and risk factors associated with having two or more chronic conditions and found that multiple chronic conditions affected nearly 10% of South Asians and each additional chronic condition carried a progressively higher risk of mortality [30]. In an Indian study conducted by Eapen et al. [24], it was found that there was an approximate 50% decrease in risk for CHD when the study participants partook in 30 to 45 min of moderate physical activity per day. Other studies have further exhibited that physical activity has had positive outcomes on abdominal adiposity, LDL (low-density lipoprotein), HDL (high-density lipoprotein), diabetes, and hypertension [24]. It is thus imperative that South Asian women engage in physical activity due to the high prevalence of metabolic syndrome and cardiovascular disease (CVD) within the population [24].

Nonetheless, South Asian women are motivated to take care of their health and bodies to decrease their risk of disease and illness [23]. Kandula et al. [25] reported that women were more likely to participate in physical activity if exercise interventions and programs were culturally appropriate, included only women, and were held in an established community setting. In addition to this, women were more motivated to engage in physical activity if children were allowed to participate [25]. Thus, it is necessary that physical activity interventions consider socio-cultural factors to ensure the effectiveness and acceptability of the intervention [25].

The importance of physical activity remains one of the least expensive and most effective preventive treatments for combating chronic disease [18]. Furthermore, research has shown the positive relationship between physical activity and mental health [31], and that physical activity, even at lower levels, has been shown to be positively associated with mental health wellbeing [32]. Sport and physical activity are powerful instruments for mobilisation and advocacy and can be used as a means for enhancing personal empowerment, combating discrimination, and achieving gender equality [19]. Increasing participation in physical activity forms a core objective across a range of government policies in most developed countries. The broad development of physical activity has become a policy target because of its significance for health care systems and economies [20].

### 1.4. Global Physical Activity Programs and Interventions

Researchers affiliated with The Waikato Institute of Technology and the University of Waikato in New Zealand focused on physical activity intervention efforts among refugee Somali women relocated to Hamilton, New Zealand [33]. Through in-person interaction with the study participants and face-to-face interviews with the women, researchers explored the barriers and intricate social and cultural dynamics involving physical activity among these refugee women [33]. From these interactions and interviews, the researchers implemented several interventions, including exercise classes and a trial-run membership to a local, women-only fitness centre [33]. While there were no data-driven indicators of the success, the intervention efforts provided researchers with viable information regarding physical activity barriers and the importance of taking a cultural approach to intervention implementation.

Wieland et al. [34], used a community-based participatory approach to analyse immigrant and refugee women in Rochester, Minnesota. In order to combat the decline in physical activity and poor nutrition intake for those who are post-immigration, researchers utilised community-based participatory research (CBPR) to create socially and culturally driven exercise and nutrition plans [34]. Forty-five women of Hispanic, Somali, Cambodian, and non-immigrant African American descent participated in this six-week program [34]. According to a physical activity class satisfaction questionnaire, upon completion of the six-week program, participants were more likely to exercise regularly, and report a higher quality of life and self-efficacy regarding diet and exercise [34]. Researchers have attributed these positive results to the socio-cultural dynamics of the program [34]. They also noted that this type of approach is more useful for refugees and immigrants displaced to Westernised settlements with no structure for maintaining diet and exercise [34].

Two additional studies utilising the CBPR approach to implement interventions include studies conducted by Marinescu et al. [35], and Dave et al. [36]. Marinescu et al. [35], utilised focus groups to evaluate and gather information regarding culture and community to specifically analyse the “Be Active Together” program directed at Muslim women in Seattle communities. This program supported physical activity and specifically provided outreach to women in public housing communities across the country [35]. From the feedback provided by the Muslim women, researchers were able to implement physical activity protocols and interventions driven by cultural insight provided by the Muslim women of these communities [35].

Dave et al. [36], interviewed South Asian immigrants in Chicago to assess physical activity barriers at various life stages. Researchers found that barriers differed depending on the life stage these women were experiencing. For older women, ailments such as chronic disease posed the largest threat to decreased physical activity [36]. In contrast, for younger women, the negative perception of being “too skinny” largely deterred these women from physical exercise [36]. In the case of women who start families or have children, researchers saw a sharp decline in physical activity, as these women saw a shift in their priorities and responsibilities to raise their families [36]. In any case, women across all ages expressed the need to consider cultural, religious, and life stages when creating physical activity interventions [36].

Another study looked at South Asian immigrant mothers at risk of type 2 diabetes in settlements located in Chicago [25]. Researchers specifically targeted women displaying risk factors for type 2 diabetes who had children between the ages of 6 and 14, in order to develop a socio-cultural physical activity intervention. The intervention ran for 16 weeks and included an exercise class two times per week, the use of Fitbits for objective exercise tracking, and educational classes on healthy eating [25]. Additionally, children were offered an exercise class alongside their mothers at the same time as their mother’s workout class [25]. The women who attended at least 80% of these workout classes lost approximately five pounds and showed a significant increase in exercise-related confidence [25]. Researchers found that this multifaceted exercise intervention structure, coupled with the cultural significance of having their children engage in physical activity alongside the mothers, was an extremely beneficial component to the intervention’s success [25].

Culturally and linguistically diverse (CALD) women from South Asia find it difficult to properly acclimate to new cultures and establish a sense of identity and belonging. Empirical evidence has found that these barriers significantly impact their health and wellbeing and limit their participation in physical activity. Due to this, South Asian women are at risk for chronic diseases, including the development of abdominal adiposity, diabetes, and CVD. However, it has been demonstrated that physical activity interventions and programs have lessened the impact of and risk for chronic diseases in this population, while also acting as positive influences on social inclusion and physical and mental health.

The main objectives of this study were:

1. Explore the beliefs, attitudes, and knowledge that South Asian women have regarding physical activity;
2. Understand the experiences that South Asian women have had when partaking in physical activity and the types of activities they engage in;
3. Understand the barriers and facilitators that South Asian women have with partaking in physical activity.

## 2. Materials and Methods

### 2.1. Context and Study

This study is the initial part of the larger SAMBA (South Asian Mothers and Children Being Active) study (2018–2021). This study was undertaken in WA and was the first stage of a larger health promotion intervention study focussing on physical activity and psychosocial wellbeing in migrant women from South Asia and the Middle East.

### 2.2. Participant Recruitment

Participants were recruited from South Asian communities through snowball and purposive sampling with the help of a key informant, who is a member of the South Asian women’s community, and with the help of already recruited South Asian women. Women who did not meet the current recommendations for physical activity requirements were targeted. Thus, women that did not meet the recommendations of 150 to 300 min of moderate-intensity physical activity or 75 to 150 min of vigorous-intensity physical activity per week outlined in Australia’s Physical Activity and Sedentary Behaviour Guidelines for Adults (18–64 years) [37], were invited to participate in the study.

### 2.3. Ethics and Informed Consent

The Curtin University Human Research Ethics Committee (HRE2018-0351) provided approval for the larger project of which this research was a component. Reciprocal ethics were obtained from the University of South Florida Institutional Review Board (Pro00035741). Participants provided informed consent to participate in the study and for their data to be used in the research. Informed consent was obtained from the participants before the focus group discussions.

### 2.4. Focus Group Discussions

This study was based on CBPR approaches and was guided by the health behaviour model [38].

Three semi-structured focus group discussions were conducted in order to assess South Asian women’s general experiences with physical activity, knowledge of physical activity, attitudes, and beliefs surrounding physical activity, as well as the facilitators, barriers, and challenges they encounter when trying to participate in physical activity. A semi-structured interview guide was used to guide the focus group discussions and the items on the guide were grounded in the health behaviour model [38]. Thus, providing rich qualitative data to further comprehend if and why challenges related to the synergistic and multifaceted relationship between health behaviours, determinants of health, as well as gender and cultural issues surrounding South Asian women have a large effect on the amounts of physical activity these women are partaking in.

A total of 16 female participants between the ages of 33 and 64 years were recruited to partake in the focus group discussions. There were four to six women per focus group. The focus groups were audio-recorded with the participants’ consent and lasted one hour to one and a half hours.

All focus group discussions were transcribed verbatim, and the qualitative data gathered from the focus groups were analysed through an inductive approach from drawing quotes and themes using thematic content analysis in order to identify and establish themes and codes [39]. The thematic analysis was conducted by the first author and the themes and codes generated were then discussed with and reviewed by the other authors. The results of this study helped guide further focus group discussions and individual interviews, which led to the development of the SAMBA physical activity intervention for CALD women.

## 3. Results

### 3.1. Demographic Data

There was a total of three focus group discussions that included a total of 16 South Asian migrant women aged between 33 and 64 years with a median age of 48 years. The focus groups included women from five of the eight countries belonging to the World Bank’s South Asia Geographic Region [40]; namely, Sri Lanka, India, Bhutan, Nepal, and Pakistan. Focus group one encompassed five women from India and one woman from Pakistan. Focus group two included six women from Sri Lanka and focus group three was comprised of four women from Nepal and one woman from Bhutan. The number of years the women have resided in Western Australia was between 4 years and 32 years, with a median period of 12 years. All the women spoke English in addition to other languages, specifically Hindi (N = 11), Marathi (N = 2), Gujarati (N = 2), Urdu (N = 1), Farsi (N = 1), Hazaraghi (N = 1), Dari (N = 1), Tamil (N = 3), Marwadi (N = 1), Punjabi (N = 1), Sinhalese (N = 4), and Nepali (N = 5). Interpreters were not needed for the focus groups.

The women had varying levels of education that included no schooling, technical school, college, or university, and post-graduate studies. There were also variations in employment status. Nine women worked full-time, where one Nepali woman stated she had her own business, three worked part-time, two identified as homemakers where one woman stated she was only on maternity leave, and two were retired. The participants came from the following religious groups: Hindu (N =8), Buddhist (N = 4), Christian (N = 2), Muslim (N = 1), and Tamil (N = 1). Furthermore, 15 of the 16 women were married, with only one woman identifying as being separated from her former husband (Table 1).

Regardless of the prior country of residence and employment status, the women across all the focus groups identified themselves as wives, mothers, homemakers, caregivers, cooks, and cleaners within their households. Many of the women stated that they lived with immediate and extended family. Those living with their immediate family stated that they lived with their husbands, children, and parents. The women living with their extended family mentioned they lived with their in-laws and/or nephews.

### 3.2. Themes

The overarching themes that were drawn from the analysis of the focus groups included the women’s (Table 2):

(1) Knowledge of physical activity with a subtheme of knowledge of frequency and duration of physical activity;
(2) General attitudes and beliefs towards physical activity, including impacts on mental health and wellbeing;
(3) Advantages and disadvantages of physical activity;
(4) Experiences with physical activity with subthemes of past physical activity, current physical activity, the difference in physical activity levels, justification for activities, preferred physical activities; and
(5) Barriers and facilitators to participating in physical activity with subthemes of barriers and challenges and facilitators.

#### 3.2.1. Theme 1: Knowledge of Physical Activity

None of the women could completely articulate the definition of physical activity. Women mentioned that physical activity involved the movement of hands and legs, an increase in breathing and heart rate, and noted that it is completed to make muscles stronger. They also mentioned that physical activity could also be relaxing and not rigorous. The examples of physical activity that the women provided included yoga, meditation, chores, mowing the lawn, walking, going to the gym, running, cycling, dancing, and Zumba.

*Subtheme 1.1: Frequency and duration.* There were some differences in how often physical activity should be completed by the participants. Some women thought physical activity should be done every day. Other women believed physical activity should be completed anywhere between two and five times a week for approximately 45 min to one hour. Interestingly, one woman explicitly stated that physical activity was not meant to be completed every day of the week. Women further mentioned that the frequency of physical activity was dependent on the age of the woman.

#### 3.2.2. Theme 2: General Attitudes and Beliefs towards Physical Activity and Wellbeing

All the women believed that physical activity was beneficial and necessary to maintain their health. They also exhibited positive attitudes toward physical activity. They understood that there were both mental and physical benefits to participating in physical activity. The women believed that physical activity was required for health and needed to be completed. All focus groups emphasised that physical activity needed to be enjoyable and fun. A woman from focus group one further explained this by stating,

*“Indian woman, Focus Group One: …I’m not learning new things. I’m just keep going doing the same thing. So, it’s all the same maybe they get bored.”*

In addition, the women stressed the importance of finding the “right exercise” to engage in. This alluded to the awareness that the frequency and types of exercise an individual can vary over the life course, especially if one is older, sick, a mother, or has experienced injury or is recovering from an injury. The women also discussed that members of their community, including friends and family, liked physical activity and believed it to be important.

#### 3.2.3. Theme 3: Advantages and Disadvantages of Physical Activity

The women acknowledged various health benefits attached to physical activity. Notably, they mentioned benefits which included taking care of their individual health, weight loss, maintaining fitness, preventing disease and heart conditions, inner happiness, flexibility, strengthening muscles, the flow of oxygen in the body, and a fresh mind. Women also stated that physical activity was good for one’s lungs, liver, breathing, and brain. Moreover, some of the women said that another benefit would be a boost in personal self-confidence. Various disadvantages of physical activity were also mentioned. These included being sore and not wanting to exercise again until the soreness subsided, as well as being too tired afterward, which impeded priorities, such as household duties, family, and cooking. A woman in focus group one specifically stated,

*“Indian woman, Focus Group One: Even if I had time, I’d rather not go for it [physical activity], because my body will ache.”*

Additional disadvantages included potential exercise-induced injuries and general pain and discomfort during and after physical activity. The women stated that other disadvantages would be over-exercising, selecting the incorrect activities to participate in—especially if one has a medical condition—and the underlying distress of not completing a physical activity in the correct way. All the women shared that physical activity provided them with improved health, increased happiness and contentment, and improved physical and mental wellbeing.

#### 3.2.4. Theme 4: Experiences with Physical Activity

During the focus groups, women were asked about their experiences with physical activity prior to migration and after migration. *Subtheme 4.1: Past physical activity.* All women in the study self-reported that they did not participate in regular physical activity, as per recommendations at the time of the study. Some of the women connected physical activity to past work-related activities and responsibilities as an employee. These activities included fieldwork and walking around an office space. In all focus groups, domesticated housework was mentioned as a type of physical activity, since the women stated it was physically taxing. The women connected physical activity to work-related activities and housework because completing these activities kept them moving with little time for sedentary behaviours.

Activities, such as walking in their home countries to get to public transport and cycling when public transport was unavailable, were also noted as past physical activities. Other past activities that the women participated in were yoga and meditation, badminton, hiking, fast-paced walking, swimming, and qigong. Using treadmills and stationary bikes at the gym were also mentioned. A woman also stated she used to participate in dance fitness classes led by an instructor but stopped because she found them too strenuous.

*Subtheme 4.2: Current physical activity.* Across all groups, walking and yoga were found to be the most prevalent types of physical activity the women were engaging in. When engaging in these activities, the duration of walking would last between 5 and 50 min, while yoga would last at least 45 min and up to 60 min. Meditation was mentioned as a type of physical activity in two groups. A woman mentioned that she line-danced two to three times per week, and another disclosed she participated in Bollywood dance classes a few times a week. Dancing and cycling were also mentioned, in addition to physical activity classes specifically tailored to older adults’ physical abilities and skills. Women mentioned that having a busy social life was part of physical activity, as it required frequent movement. One woman stated that she recently joined a gym and was trying to attend regularly.

*Subtheme 4.3: Differences in physical activity level.* Across all groups, most of the women mentioned a decrease in activity when they compared their current physical activity level to their past activity level. Women stated that age was an influential factor for varying levels of physical activity. These women expressed that, as women age, physical activity decreases overall, especially high-intensity activities because the body changes with age and health issues and complications can present. Therefore, these factors generally decrease participation in an activity and the level at which a woman can sustain an activity for an extended period. Women in two discrete groups agreed that they were more active in their home countries since they either did not have access to a personal vehicle or had limited access to public transportation for travel. This required them to walk more, which inherently increased their frequency and duration of physical activity. A woman also mentioned that walking in Australia is less strenuous than it was in her home country because the weather reached higher temperatures, which led to discomfort.

*Subtheme 4.4: Justifications for activities.* Many reasons were discussed as to why the study participants were partaking in their current activities. Women stated that they engaged in their current physical activities to benefit their physical and mental health. They also mentioned that they participated in the activities because they wanted to look good in regard to body image. Across all groups, the idea of social support was given as a reason for partaking in physical activity. Participants found that completing activities with family or friends in a group setting was easier, more fun, and more enjoyable than doing physical activity alone. There was also a sense of increased self-efficacy when completing activities in groups. To elaborate on self-efficacy, one woman stated,

*“Indian woman, Focus Group One: …there are a bunch of mums who have kids and they’re able to do, why not me??”*

Women also mentioned that the activities they participated in were convenient for them and that members of their family encouraged them to engage in those specific physical activities. Women in another group discussed that age, as well as injuries and pain, were decisive factors in terms of the activities they chose to participate in. Women in this group also expressed that engaging in their physical activities made them ‘feel good’ and less irritable, improved their mental health, helped with weight loss, and empowered them. Women in another group voiced how they believed the activities they engaged in were the safest activities for them, fit within cultural boundaries and appropriateness, and were also easy to perform and matched their abilities. To further explain this, one woman stated,

*“Nepali woman, Focus Group Three: I would not run, because I cannot run for long and cannot run far. So, I would not do it, and it makes me self-conscious since I cannot do it well.”*

Women also discussed how they had become comfortable with the activities that they are completing, due to repeated participation in the activity.

*Subtheme 4.5: Preferred physical activities.* Women preferred to participate in low-intensity exercises and activities, such as walking and yoga. In contrast, they were less inclined to participate in activities that were perceived as being rigorous or complex. The focus groups specifically yielded qualitative information stating that the women would not participate in activities utilising dumbbell weights, because the activity was too rigorous, and they did not want to be in pain or injure themselves.

*“Indian woman, Focus Group One: Oh, I would least likely do those dumbbells, and other rigorous activities, I can’t do it.”*

The women also stated they would not participate in vigorous running and jogging because they did not want to risk injury. Women also mentioned that they were less likely to participate in swimming for physical activity than other types of physical activity. They stated that they felt insecure about their underdeveloped level of skill and expressed fears of drowning. This was compounded by the idea that they believed it was too late in life for them to learn new or unfamiliar skills to carry out certain activities.

*“Sri Lankan woman, Focus Group Two: I’m too old to learn how to swim you need to get those skills when you are younger. It’s harder to learn properly when you are older.”*

Another woman also expressed her battle with her skill level when discussing participation in fitness classes she attended, which can be linked to feelings of inadequacy.

*“Indian woman, Focus Group One: …I don’t like [name of dance-based fitness class] and aerobics. I’m not coordinated, my body is not made for that. I tried, it just doesn’t work for me.”*

#### 3.2.5. Theme 5: Barriers, Challenges, and Facilitators Related to Participation in Physical Activity

The barriers and challenges faced by the migrant women from South Asia in regard to taking part in physical activities were explored in this theme. *Subtheme 5.1: Barriers and challenges.* Across all groups, time, cost, and lifestyle factors, including stress, were expressed as barriers and challenges. Participants felt that there was not enough time in the day to adequately participate in physical activity. Most of the women were employed full-time, and were also primarily responsible for activities, including tending to children and the house and cooking meals for the family. Participants also gave priority to frequent community and cultural activities, many of which do not traditionally involve physical activity. In addition, women also stated that not having a group of friends or social network to complete a physical activity with, soreness, and the presence of men while engaging in physical activity were all barriers and challenges that made it difficult for them to participate in physical activity. Women in two discrete groups expressed tiredness and a lack of motivation to engage in physical activity as challenges. Similarly, one woman stated she would rather prioritise sleep than do physical activity.

All focus groups also identified fear of judgement from others, their personal insecurities, shyness, self-consciousness, and embarrassment surrounding their skills and abilities for physical activity as barriers to participation and expressed how this impacted their personal wellbeing. This is best presented through the words of one woman,

*“Indian woman, Focus Group One: I don’t know if it’s my own insecurity, like I’m being judged every time I take a wrong step. I feel like I’m being judged for that, which I’m sure people don’t care. They’re not even looking at you. I think it’s just a built-in insecurity really are they looking?”*

Facilitators that help women make informed decisions about physical activity were also examined. *Subtheme 5.2: Facilitators.* In order to make physical activity easier and not burdensome for South Asian women to complete, a variety of factors need to be considered. Participants stated that a physical activity class or facility located near their places of residence with group classes later in the evening would be extremely beneficial and convenient. Participants explained that this would allow them to complete their household tasks and outstanding priorities before engaging in physical activity and becoming tired since energy is needed to fulfil daily work, cooking, and caretaking. It would also allow them to take part in physical activity with friends and other migrant women, developing social support and group motivation. Furthermore, an affordable gym or recreation facility membership or free services would encourage women to participate in physical activity more often. One group stated that women-only facilities or classes would be advantageous and accommodating for reasons related to culture, as well as self-consciousness. It was also discussed that low-cost or free childcare would be beneficial because a woman’s husband is traditionally tired after working a long day. Therefore, the women do not want to concern the husband or other members of the family with childcare. The idea of allowing children to attend physical activity classes alongside their mothers also emerged, was positively accepted, and thought of as fun.

## 4. Discussion

This qualitative study drew on the experiences and knowledge of South Asian migrant women residing in Western Australia. The thematic analysis of focus group discussions found that the South Asian women included in this study face various internal and external influences when participating in physical activity. Relevant topics discussed during the discussions included the women’s knowledge of physical activity, their general attitudes and beliefs about physical activity, advantages and disadvantages of physical activity, experiences with physical activity, and the barriers, challenges, and facilitators surrounding physical activity.

Notably, the barriers that these women faced can be described as determinants of health that are embedded within the levels of the social-ecological model, specifically the individual, interpersonal, organisational, and community levels. Interestingly, even though there was a diverse sample of women from five South Asian countries, all the women discussed similar socio-cultural influences to participating in physical activity. Thus, solidifying that the influences, which reside within the social-ecological model of health, can persist in various CALD populations.

On comparing the results of this study to previous literature [5,6,23,33,35], we identified several themes that CALD migrant women populations face. Participants in this study understood and valued the importance of the positive association between physical activity and physical and mental health wellbeing and identified various personal, social, and environmental barriers and facilitators to participation. Influences of participation in physical activity, found in this study and also presented and discussed in previous research, included work, culture, gender, cost, time, family, health status, and environmental factors [41,42]. However, the participants in this study did not find language, religion, dress, or social isolation as influencing their participation in physical activity.

### 4.1. Strengths and Limitations

A limitation of the study is the small and heterogeneous sample size. Therefore, the results presented are not generalizable to other CALD populations. However, being a qualitative study, depth, transferability, and credibility were of importance. A strength of this study was providing South Asian migrant women with a voice to share their thoughts about physical activity and wellbeing. The sharing of experiences revealed challenges and successes the women had faced during resettlement and provided rich data on what physical activity program could be designed and would interest the women. Interviewer bias may be another limitation of this study. In order to avoid this bias, the focus groups were semi-structured, and prompts were frequently utilised after multiple questions to stimulate discussion. Future studies should improve by recruiting a larger sample size that includes women from the South Asian countries that were not represented in this study. In addition, matching the number of participants from each country between groups could increase the generalizability of the results, something that was not possible in this study due to time constraints in recruitment. For future studies, the sample size could be expanded to women who also meet the weekly physical activity recommendations to allow for comparisons and identify factors of participation. Individual interviews, rather than focus group discussions, may reflect country contexts and cultural differences better and allow for a more in-depth exploration of the physical activity experiences of South Asian women.

### 4.2. Recommendations

Failing to address socio-cultural influences within this population significantly hinders the population’s ability to partake in physical activity. Therefore, based on this study, it is recommended that targeted and sustained funding for future programs and interventions targeting this population include an educational component. Including an educational component in a program or intervention would dispel any confusion concerning the recommended duration and frequency of physical activity, and would describe the benefits of physical activity, and address age-appropriate exercise, provide strategies for motivation and sustained participation in physical activity, as well as provide information on activities for women who have underlying health conditions. Providing the women with opportunities to increase their knowledge also has the potential to lead to individual and group empowerment and increase motivation and self-efficacy. Similarly, the study documented that the women are capable of learning new skills, even later in life.

Additionally, physical activity programs should be made fun and enjoyable for the women versus being constructed as rigorous and repetitive. A program’s emphasis on women-only group classes would likely result in higher and sustained engagement instead of constructing a program to cater to individual-level participation in physical activity. Furthermore, group classes should be held at a mutually convenient time and close to the women’s homes. Allowing children to participate in physical activity alongside their mothers would also be beneficial because it would be more fun for the women, and they would not need to find supplemental childcare. Finally, it is recommended that CBPR methods are employed as an effective means to further consider the social, cultural, and environmental determinants of health when implementing public health measures to improve physical and mental health and wellbeing for populations globally.

CBPR is increasingly utilised in public health to connect educational opportunities with social action, especially regarding chronic disease prevention [43,44,45]. CBPR offers the opportunity for public health professionals to engage with communities to further understand complexities on a local level within populations and complexities on a macro-level, which are often overlooked but contribute to unsustainable interventions and programs [45]. Since CBPR engages the community, beneficiaries, and stakeholders, while considering a community’s knowledge, attitudes, beliefs, and cultural practices, it also ensures that the objectives and goals of a study are sensible, relevant, and consistent with the target population’s needs [46].

CPBR offers the ability to address health disparities and inequities, as well as environmental and social justice [43,47]. Subsequently, this has empowered communities to take charge of the determinants affecting their health and form community coalitions [45]. Therefore, it is expected that interventions and programs utilising CBPR will elicit greater positive impacts, long-term benefits, reduce community resistance to participation, and meet a community’s needs [47].

The results of this study helped guide further focus group discussions and individual interviews, which led to the development of the SAMBA physical activity intervention using CBPR methods. The SAMBA study was a pilot physical activity intervention study conducted with CALD women from South Asian and Middle Eastern backgrounds in Western Australia.

## 5. Conclusions

This study contributes to prior research and uncovers new findings within the Western Australian context and challenges related to the synergistic and multifaceted relationship between health behaviours, determinants of health, as well as gender and cultural factors surrounding South Asian migrant women and physical activity. Specifically, it exemplifies that physical activity participation within the South Asian women population in Western Australia is low. It additionally provides insights into the various influences that affect the participation rates of migrant South Asian women in physical activity. Therefore, this study offers important socio-cultural information for the development of successful and sustainable physical activity interventions for South Asian women. By providing formative research for the development of culturally sensitive physical activity programs, which can ultimately contribute to higher physical activity participation rates and a greater quality of life for this population, can, in turn, improve the mental health and physical wellbeing for migrant women and their families.

## Figures and Tables

**Table 1:** Demographic characteristics of study participants.

Characteristic	Count
Age in Years	N
32–38	3
39–45	3
46–52	7
53–59	1
60–66	2
Previous Country of Residence	N
Sri Lanka	5
India	5
Bhutan	1
Nepal	4
Pakistan	1
Number of Years in Western Australia	N
1–5	2
6–10	5
11–15	2
16–20	3
21–25	1
26–30	1
31–35	2
Education	N
No School	1
Technical school	2
College or University	9
Post-Graduate	4
Employment Status	N
Full-Time	9 *
Part-Time	3
Homemaker	2 **
Retired	2
Marital Status	N
Married	15
Separated	1

* Including one participant who owns a business with her husband ** Including one participant who is on maternity leave.

**Table 2:** Table of themes drawn from the analysis.

Themes	Subthemes
Knowledge of physical activity	Frequency and duration of physical activity
General attitudes and beliefs toward physical activity	
Advantages and disadvantages of physical activity	
Experiences with physical activity	Past physical activity Current physical activity Difference in physical activity levels Justifications for activities Preferred physical activities
Barriers, challenges, and facilitators surrounding physical activity	Barriers and challenges Facilitators

## Data Availability

The datasets produced and analyzed for this study are available from the corresponding author on reasonable request.
